# EHMTI-0085. White matter microstructure abnormalities in pediatric migraine patients: in-vivo measures of brain hyperexcitability?

**DOI:** 10.1186/1129-2377-15-S1-E18

**Published:** 2014-09-18

**Authors:** R Messina, MA Rocca, B Colombo, E Pagani, A Falini, G Comi, M Filippi

**Affiliations:** 1 Neuroimaging Research Unit and Dept. of Neurology, Scientific Institute and University Hospital San Raffaele, Milan, Italy; 2 Dept. of Neurology, Scientific Institute and University Hospital San Raffaele, Milan, Italy; 3 Dept. of Neuroradiology, Scientific Institute and University Hospital San Raffaele, Milan, Italy

## Introduction

By exploiting diffusion characteristics of water molecules in the central nervous system, diffusion tensor (DT) magnetic resonance imaging (MRI) provides several quantities with the potential to disclose WM microstructure abnormalities. Among these, fractional anisotropy (FA) reflects axonal integrity and fiber organization, mean diffusivity (MD) measures the overall magnitude of diffusion, axial diffusivity (AD) is associated with fiber density and axon intrinsic characteristics, whereas radial diffusivity (RD) reflects the degree of myelination.

## Aims

To explore abnormalities of white matter (WM) microstructure in pediatric patients with migraine using DT MRI and two different methods of analysis.

## Methods

Using a 3.0 Tesla scanner, dual-echo and DT MRI scans were acquired from 15 pediatric migraine patients and 15 age-matched controls. Tract-based spatial statistics (TBSS) analysis was performed using FMRIB's Diffusion Toolbox. In order to confirm TBSS results, we also performed a DT probabilistic tractography analysis.

## Results

Both TBSS and DT tractography analysis showed that compared to controls, pediatric migraine patients had significantly lower MD, AD and RD of the brainstem, thalamus and fronto-temporo-occipital lobes. They also experienced increased FA of the left optic radiation. No correlation was found between WM abnormalities and disease duration and frequency of attacks.

## Conclusions

Pediatric patients with migraine experience diffuse WM microstructural abnormalities. Higher FA and lower MD, AD and RD might be explained by repeated neuronal activation or by the presence of higher neuronal and synaptic densities in these patients compared to controls. Both these mechanisms would reflect a hyperexcitability of the brain in migraine patients.

No conflict of interest.

